# FACTORS CONTRIBUTING TO PULMONARY TB TREATMENT LOST TO FOLLOW-UP IN DEVELOPING COUNTRIES: AN OVERVIEW

**DOI:** 10.21010/Ajidv17i1.6

**Published:** 2022-12-22

**Authors:** OPPERMAN, Monique, DU PREEZ, Ilse

**Affiliations:** Human Metabolomics, North-West University (Potchefstroom Campus), Private Bag x6001, Box 269, Potchefstroom, South Africa, 2531

**Keywords:** Developing countries, DOTS adherence, Adherence interventions, Lost to follow-up, Tuberculosis

## Abstract

**Background:**

Despite the available treatment options, pulmonary tuberculosis (TB) remains a leading cause of disease-related deaths worldwide. Treatment non-adherence/lost to follow-up (LTFU), particularly in developing countries, is a continuous concern. LTFU prolongs TB infectiousness and contributes to TB treatment failure, relapse, and death. Furthermore, LTFU also delays global TB eradication by promoting TB spread and drug-resistant TB strain development.[1] The purpose of this paper is to give an overview of the commonly observed risk factors associated with TB treatment LTFU in developing countries.

**Materials and Methods:**

A literature survey was done of studies published in the past decade, which evaluated the risk factors for LTFU in TB patients, specifically in developing countries. Furthermore, some prospective TB treatment adherence initiatives and the feasibility of these initiatives within developing countries were assessed.[3]

**Results:**

Several variables, including socio-demographic, patient-related, TB disease and other health-related-factors, healthcare and system determinants, as well as treatment-related factors, were identified to increase the risk of TB treatment LTFU. More recently applied adherence interventions in developing countries, show potential for implementation on a larger scale.

**Conclusion:**

Successful TB treatment is contingent on treatment adherence, and by addressing these persisting LTFU risk factors, treatment adherence in developing countries may be improved.

## Introduction

Pulmonary tuberculosis (TB) is currently the second leading cause of death from a single infectious agent, following coronavirus disease (COVID-19), and ranking above human immunodeficiency virus (HIV)/acquired immune deficiency syndrome (AIDS) (WHO, 2021). In 2020 alone, an estimated ten million people globally were newly infected with the causative agent of TB, *Mycobacterium tuberculosis (M. tuberculosis)* (WHO, 2021). Developing countries, broadly located in Africa, Latin America, the Caribbean, Asia (excluding Israel, Japan, and South Korea) and Oceania (excluding Australia and New Zealand) (UNCTAD, 2022) are mostly affected, with TB incidence rates of up to more than 500 cases per 100 000 population per year, as opposed to less than 10 cases per 100 000 population per year in developed countries (WHO, 2021). Furthermore, 84% of all reported TB deaths globally (among HIV-negative individuals) occurred in the African and South-East Asia regions in 2019 (WHO, 2021).

TB is, however, considered a curable disease, with effective treatment regimens in place. In 2019, treatment success rates (including cured and treatment completed outcomes) were reported to be 86% for new and relapse TB cases, 77% for new and relapse HIV-positive TB cases, and 59% for multidrug-resistant and rifampicin-resistant cases (WHO, 2021). The unsuccessful treatment cases cumulatively include failed treatment, deceased cases, those not evaluated, and lost to follow-up patients (LTFU) (Table 1), the latter being the focus of this review.

**Table 1:** World Health Organisation (WHO)-assigned definitions for treatment outcomes of Tuberculosis patients (excluding multidrug- or rifampicin-resistant cases) (WHO, 2014**)*.***

Tuberculosis treatment Outcome	*World Health Organisation (WHO)*-assigned definition
Cured	A pulmonary TB patient with bacteriologically confirmed TB at the beginning of treatment who was smear- or culture-negative in the last month of treatment and on at least one previous occasion.
Treatment completed	A TB patient who completed treatment without evidence of failure BUT with no record to show that sputum smear or culture results in the last month of treatment and on at least one previous occasion were negative, either because tests were not done or because results are unavailable.
Treatment failed	A TB patient whose sputum smear or culture is positive at month five or later during treatment.
Died	A TB patient who dies for any reason before starting or during the course of treatment.
Lost to follow-up (LTFU)	A TB patient who did not start treatment or whose treatment was interrupted for two consecutive months or more.
Not evaluated	A TB patient for whom no treatment outcome is assigned. This includes cases “transferred out” to another treatment unit as well as cases for whom the treatment outcome is unknown to the reporting unit.
Treatment success	The sum of cured and treatment completed.

Given the adaptability of bacteria to the environment, adherence to the prescribed TB treatment regimen is crucial, and even one missed dose greatly increases the chance of acquiring drug-resistant *M. tuberculosis* (Tola *et al*., 2019). In addition to the development of drug resistance, treatment LTFU can also cause prolonged infectiousness, with the possibility of treatment failure, relapse, and death (Tola *et al.*, 2019).

TB treatment LTFU can be a consequence of a large number of variables, which can be divided into five categories, namely: (1) socio-demographic factors, (2) patient-related factors, (3) TB disease and other health-related factors, (4) healthcare and system determinants, and (5) treatment-related factors (WHO, 2003).

Despite the acknowledgement of these LTFU risk factors, treatment non-adherence persists, particularly in developing countries such as Ethiopia and Nigeria, with TB LTFU rates in 2021 being reported as 20.9% (Kimani *et al.*, 2021) and 30.5% (Iweama *et al.*, 2021), respectively.

The focus of this review is to give an overview of the risk factors which are regularly associated with first-line TB treatment LTFU in developing countries, and to evaluate if these factors still match those determined previously (WHO, 2003).

Alternative TB treatment regimens and drug resistance can have an impact on LTFU incidence. Therefore, we mainly focussed on the LTFU determinants of drug-susceptible pulmonary TB cases, treated under the standardised first-line treatment regime in developing countries. Furthermore, we assess the efficacy of recently proposed adherence intervention strategies, with a specific focus on their feasibility in developing countries.

### Factors associated with TB treatment lost to follow-up

### Socio-demographic factors

Treatment adherence is known to be influenced by socio-demographic characteristics such as age, gender, nationality, race, occupation, residential area, education level, monthly income, marital status, and socioeconomic stand (WHO, 2003). The following is an overview of the most prevalent socio-demographic factors currently associated with TB treatment LTFU in developing countries.

### Age

The occurrence of age as a risk factor for TB treatment non-adherence is contradictory. Several studies have found that older age (ranging from >45 to >60 years) is associated with TB treatment LTFU (Chen *et al.*, 2013, Patra *et al.*, 2013, Lin *et al.*, 2017, Mukhtar and Butt, 2017, Mundra *et al.*, 2017, Muluye *et al.*, 2018), while others linked this risk to younger age groups, mostly between 15 and 45 years (Marx *et al.*, 2012, Nour El Din *et al.*, 2013, Nglazi *et al.*, 2015, Tesfahuneygn *et al.*, 2015, Harling *et al.*, 2017, Wohlleben *et al.*, 2017, Mulongeni *et al.*, 2019, Kimani *et al.*, 2021). Some studies, on the contrary, found that age had no significant impact on TB treatment adherence, including more recent research conducted in several developing countries (Ajema *et al.*, 2020, Robsky *et al.*, 2020, Ahmed and Mohan, 2021, Iweama *et al.*, 2021, Workie *et al.*, 2021).

The rationale behind age group as a risk factor for treatment LTFU was previously related to the fact that older patients experience more extensive TB disease or other age-related health comorbidities (Mundra *et al.*, 2017) and suffer more severe adverse drug events (Chida *et al.*, 2015). Younger age groups, on the other hand, were more likely to be subjected to additional socioeconomic obstacles, such as work responsibilities (Chida *et al.*, 2015, Kimani *et al.*, 2021). These characteristics are, however, not set in stone, and age should therefore be considered as a risk factor on an individual basis, taking other related factors and circumstances into account.

### Gender

Although gender is rarely identified as a significant treatment LTFU risk factor (Peltzer *et al.*, 2012, Sendagire *et al.*, 2012, Zhou *et al.*, 2012, Jenkins *et al.*, 2013, Kayigamba *et al.*, 2013, Liddle *et al.*, 2013, Nour El Din *et al.*, 2013, Babiarz *et al.*, 2014, Putera *et al.*, 2015, Roy *et al.*, 2015, Tesfahuneygn *et al.*, 2015, Theron *et al.*, 2015, Ali and Prins, 2016, Flick *et al.*, 2016, Mukhtar and Butt, 2017, Viegas *et al.*, 2017, Woimo *et al.*, 2017, Ambaw *et al.*, 2018, Mekonnen and Azagew, 2018, Fang *et al.*, 2019, Ajema *et al.*, 2020, Robsky *et al.*, 2020, Iweama *et al.*, 2021, Workie *et al.*, 2021), some studies have stipulated that being male increased the risk of TB treatment non-adherence (Muture *et al.*, 2011, Culqui *et al.*, 2012, Garrido *et al.*, 2012, Marx *et al.*, 2012, Babalik *et al.*, 2013, Naidoo *et al.*, 2013, Tachfouti *et al.*, 2013, Babalik *et al.*, 2014, Cherkaoui *et al.*, 2014, Chida *et al.*, 2015, Lackey *et al.*, 2015, Nglazi *et al.*, 2015, Lei *et al.*, 2016, Harling *et al.*, 2017, Kigozi *et al.*, 2017, Lin *et al.*, 2017, Mundra *et al.*, 2017, Silva *et al.*, 2017, Wohlleben *et al.*, 2017, Gube *et al.*, 2018, Muluye *et al.*, 2018, Mulongeni *et al.*, 2019, Afshari *et al.*, 2020). This might, in turn, be connected to the increased TB prevalence in males, with adult males accounting for 56% of all newly reported TB cases in 2020 (WHO, 2021). Additionally, it was suggested that this could be linked to other socio-economic factors, such as, for example, the fact that males are often seen as the primary source of income in many cultures and cannot afford to cease working to get regular medical care (Tachfouti *et al.*, 2013).

### Socio-economic stand

Income, education, and employment are all elements which have an impact on an individual’s ability to make healthy decisions and could therefore add to the prevalence of TB treatment LTFU. In accordance, numerous independent studies identified factors related to a poor socio-economic stand as being determinants of TB treatment LTFU. This included low monthly income (Muture *et al.*, 2011, Cherkaoui *et al.*, 2014, Theron *et al.*, 2015, Gube *et al.*, 2018, Fang *et al.*, 2019, Ahmed and Mohan, 2021, Iweama *et al.*, 2021), rural (Ali and Prins, 2016, Lin *et al.*, 2017, Mukhtar and Butt, 2017) or urban residence area (Tachfouti *et al.*, 2013, Ambaw *et al.*, 2018), dwelling type such as homelessness, shared/sheltered housing (Peltzer *et al.*, 2012, Jenkins *et al.*, 2013, Cherkaoui *et al.*, 2014, Silva *et al.*, 2017, Wohlleben *et al.*, 2017), unemployment (Tachfouti *et al.*, 2012), blue collar type of work (Finlay *et al.*, 2012, Liddle *et al.*, 2013, Ali and Prins, 2016, Woimo *et al.*, 2017) or work that require long working hours (Zhou *et al.*, 2012, Cherkaoui *et al.*, 2014, Mekonnen and Azagew, 2018), and low education levels (Culqui *et al.*, 2012, Finlay *et al.*, 2012, Garrido *et al.*, 2012, Jenkins *et al.*, 2013, Cherkaoui *et al.*, 2014, Lackey *et al.*, 2015, Tesfahuneygn *et al.*, 2015, Mukhtar and Butt, 2017, Silva *et al.*, 2017, Woimo *et al.*, 2017, Gube *et al.*, 2018).

### Patient-related factors

Patient-related factors, such as, for example, social support, TB disease and treatment perceptions and knowledge, TB stigmatisation, as well as other behavioural factors, including relocation, travelling, smoking, alcohol, and substance use, have been shown to influence an individual’s health-seeking behaviours and TB treatment adherence.

### Lack of social support

The lack of social support, defined as the perceived and actual lack of assistance given by close friends, family, and the community (Zarova *et al.*, 2018, Chen *et al.*, 2021), have been identified as a risk factor for LTFU in developing countries (Culqui *et al.*, 2012, Finlay *et al.*, 2012, Liddle *et al.*, 2013, Cherkaoui *et al.*, 2014, Ali and Prins, 2016, Gube *et al.*, 2018, Ajema *et al.*, 2020). Patients who have sufficient social support report greater levels of physiological well-being, while those who lack social support could experience a worse quality of life and higher levels of physiological stress. This in turn, have been linked to patients being less likely to adhere to TB treatment (Zarova *et al.*, 2018, Chen *et al.*, 2021).

### TB disease and treatment illiteracy

Limited knowledge about the TB disease and the treatment thereof amongst populations in developing countries is also frequently identified as a significant factor contributing towards LTFU (Vijay *et al.*, 2010, Muture *et al.*, 2011, Tachfouti *et al.*, 2012, Zhou *et al.*, 2012, Cherkaoui *et al.*, 2014, Putera *et al.*, 2015, Roy *et al.*, 2015, Theron *et al.*, 2015, Viegas *et al.*, 2017, Woimo *et al.*, 2017, Mekonnen and Azagew, 2018, Fang *et al.*, 2019, Ajema *et al.*, 2020, Iweama *et al.*, 2021). This treatment illiteracy leads to many patients simply stopping treatment, either because they felt some discomfort (adverse drug events) during treatment (Vijay *et al.*, 2010, Muture *et al.*, 2011, Finlay *et al.*, 2012, Sendagire *et al.*, 2012, Tachfouti *et al.*, 2012, Cherkaoui *et al.*, 2014, Chida *et al.*, 2015, Roy *et al.*, 2015, Tesfahuneygn *et al.*, 2015, Wohlleben *et al.*, 2017, Woimo *et al.*, 2017, Gube *et al.*, 2018, Iweama *et al.*, 2021) or started to feel better (Muture *et al.*, 2011, Peltzer *et al.*, 2012, Sendagire *et al.*, 2012, Tachfouti *et al.*, 2012, Liddle *et al.*, 2013, Cherkaoui *et al.*, 2014, Ahmed and Mohan, 2021, Kimani *et al.*, 2021), without comprehending the full consequences of treatment interruption. A study conducted in India found that the majority of the LTFU patients investigated seem to complete at least the first two months of treatment (Ahmed and Mohan, 2021), while in a South African study it was found that the median period from treatment onset to LTFU was 101 days which, in turn, was linked to TB symptom relief (Kigozi *et al.*, 2017). In a few instances, however, particularly in studies conducted in Ethiopia and India, the association between TB and treatment illiteracy and LTFU incidences was found to be meaningless (Tesfahuneygn *et al.*, 2015, Workie *et al.*, 2021).

### TB stigmatisation

TB and treatment illiteracy are further enhanced by misinformation, which according to Msoka *et al*. (2021) creates a knowledge gap that is often filled with superstition and stigma. In West Africa for instance, Msoka *et al*. (2021) reported a stigma that links TB with an ethnic or familial curse, and it is thought that patients are extremely unhygienic, and that the disease is highly contagious, so much so that even healthcare practitioners avoid interacting with TB patients. As a result, individuals are more likely to keep their disease hidden and are less inclined to seek medical care (Msoka *et al.*, 2021). Furthermore, it is reported that the general community in East Africa believe that anybody testing positive for TB is also HIV infected, exacerbating unfavourable attitudes toward TB patients, and negatively impacting treatment adherence (Msoka *et al.*, 2021).

Although not classified as a risk factor for LTFU in a number of studies (Tachfouti *et al.*, 2012, Gube *et al.*, 2018, Ajema *et al.*, 2020, Iweama *et al.*, 2021, Workie *et al.*, 2021), several studies did identify TB stigmatisation as a significant treatment LTFU risk factor (Muture *et al.*, 2011, Finlay *et al.*, 2012, Nour El Din *et al.*, 2013, Putera *et al.*, 2015, Wohlleben *et al.*, 2017). In contrast, however, one study conducted in South Korea, a developed country, reported that TB stigmatisation enhances treatment adherence and identified stigma absence as a predictor of treatment LTFU (Park *et al.*, 2016). This occurrence was, however, still attributed to the patients’ shame for having tuberculosis and thus seeking treatment early (Park *et al.*, 2016).

### Relocation and travelling

Different investigations could associate relocation (Finlay *et al.*, 2012, Peltzer *et al.*, 2012, Sendagire *et al.*, 2012, Ali and Prins, 2016, Mukhtar and Butt, 2017, Wohlleben *et al.*, 2017, Kimani *et al.*, 2021) and travelling (Liddle *et al.*, 2013, Tesfahuneygn *et al.*, 2015, Mekonnen and Azagew, 2018, Kimani *et al.*, 2021) during TB treatment with an increased risk of treatment non-adherence. This was mostly ascribed to the fact that patients are not aware of procedures for treatment transfers to other facilities or drug collection while travelling.

### Alcohol, smoking and substance use

In addition to the factors discussed above, other social behavioural factors, such as excessive tobacco, alcohol, and substance use, have been shown to contribute to TB treatment LTFU.

Alcohol consumption, ranging from moderate (Culqui *et al.*, 2012, Finlay *et al.*, 2012, Cherkaoui *et al.*, 2014, Lackey *et al.*, 2015, Roy *et al.*, 2015, Harling *et al.*, 2017, Gube *et al.*, 2018, Mekonnen and Azagew, 2018) to heavy (Vijay *et al.*, 2010, Muture *et al.*, 2011, Garrido *et al.*, 2012, Sendagire *et al.*, 2012, Jenkins *et al.*, 2013, Naidoo *et al.*, 2013, Tesfahuneygn *et al.*, 2015, Theron *et al.*, 2015, Silva *et al.*, 2017, Viegas *et al.*, 2017, Workie *et al.*, 2021) alcohol use, is found to be significantly associated with TB LTFU cases. However, there are a number of studies where this relationship was considered to be insignificant (Peltzer *et al.*, 2012, Nour El Din *et al.*, 2013, Putera *et al.*, 2015, Wohlleben *et al.*, 2017, Woimo *et al.*, 2017, Ambaw *et al.*, 2018, Ajema *et al.*, 2020, Iweama *et al.*, 2021).

In a similar manner, many studies point to current smoking or tobacco use as a significant risk factor for TB LTFU (Vijay *et al.*, 2010, Naidoo *et al.*, 2013, Tachfouti *et al.*, 2013, Cherkaoui *et al.*, 2014, Chida *et al.*, 2015, Roy *et al.*, 2015, Tesfahuneygn *et al.*, 2015, Mukhtar and Butt, 2017, Gube *et al.*, 2018, Ajema *et al.*, 2020, Iweama *et al.*, 2021, Workie *et al.*, 2021), with a few identifying smoking history as a contributing element (Lackey *et al.*, 2015, Silva *et al.*, 2017). On the contrary, some studies found no significant association between smoking status and treatment LTFU (Peltzer *et al.*, 2012, Nour El Din *et al.*, 2013, Putera *et al.*, 2015, Theron *et al.*, 2015, Viegas *et al.*, 2017).

Many research groups additionally linked substance abuse with TB treatment LTFU (Culqui *et al.*, 2012, Jenkins *et al.*, 2013, Cherkaoui *et al.*, 2014, Lackey *et al.*, 2015, Tesfahuneygn *et al.*, 2015, Silva *et al.*, 2017, Wohlleben *et al.*, 2017, Ambaw *et al.*, 2018), with only a few reporting this association to be insignificant (Finlay *et al.*, 2012, Ajema *et al.*, 2020, Workie *et al.*, 2021).

These behavioural actions could lead to the disruption of medication intake (i.e., lead to forgetfulness (Roy *et al.*, 2015)) and negatively affect help-seeking and treatment processes, resulting in poor treatment adherence (Theron *et al.*, 2015, Workie *et al.*, 2021). Alcohol use for instance, has been linked to increased physiological stress, which in turn has also been associated with TB treatment non-adherence (Theron *et al.*, 2015). Furthermore, alcohol use also contributes to liver damage, which could aggravate the adverse drug events induced by TB drugs (Roy *et al.*, 2015, Theron *et al.*, 2015).

### Healthcare and system determinants

In addition to variables related to the patients themselves, treatment adherence is also influenced by factors related to healthcare and the systems in place to provide TB treatment. These determinants include: the accessibility to treatment sites, patient-provider interactions, treatment costs, healthcare worker expertise and TB and treatment counselling given, the sort of DOT (directly observed treatment) supervision, treatment costs and diagnostic regimes.

### TB treatment site accessibility

When assessing characteristics associated with TB treatment LTFU, specifically in developing countries, accessibility to TB treatment sites is a crucial issue to consider. For many TB patients, DOT clinics are too far away, and transit costs can be prohibitively high, if at all available (Woimo *et al.*, 2017). Although the relationship between the location of the treatment site and LTFU incidence is occasionally shown to be negligible (Vijay *et al.*, 2010, Zhou *et al.*, 2012, Roy *et al.*, 2015, Tesfahuneygn *et al.*, 2015, Harling *et al.*, 2017, Fang *et al.*, 2019), it is frequently indicated to be significant (Tachfouti *et al.*, 2012, Ali and Prins, 2016, Lei *et al.*, 2016, Woimo *et al.*, 2017, Gube *et al.*, 2018, Mekonnen and Azagew, 2018, Iweama *et al.*, 2021), with patients often citing long distance (Nour El Din *et al.*, 2013) and inaccessibility (Muture *et al.*, 2011) to treatment sites as reasons for LTFU. Interestingly, a Ugandan study found that being two kilometres away from the treatment site, as opposed to being closer, resulted in better treatment adherence (Robsky *et al.*, 2020). These patients did, however, present with a greater risk of mortality during treatment due to more severe TB disease, which is suggested to have driven them to better adhere to treatment (Robsky *et al.*, 2020).

### Patient-provider relationship and perceived healthcare worker expertise/attitude

Several LTFU patients have reported that they did not receive appropriate care or support during their treatment and that this led to the consequent non-adherence. Inconvenient clinic hours (Finlay *et al.*, 2012), long waiting times (Muture *et al.*, 2011, Nour El Din *et al.*, 2013, Tesfahuneygn *et al.*, 2015, Gube *et al.*, 2018, Workie *et al.*, 2021), and poor patient-provider relationships (due to patient perceived attitude/trustworthiness of healthcare staff (Vijay *et al.*, 2010, Culqui *et al.*, 2012, Tachfouti *et al.*, 2012, Nour El Din *et al.*, 2013, Roy *et al.*, 2015, Woimo *et al.*, 2017, Mekonnen and Azagew, 2018)) are frequently mentioned by patients in developing countries as reasons for LTFU. Furthermore, numerous patients stated that they received insufficient education/counselling about TB treatment and that the rationale behind the six-month program was not explained (Finlay *et al.*, 2012, Zhou *et al.*, 2012, Lei *et al.*, 2016, Woimo *et al.*, 2017).

### DOT supervision

The World Health Organisation (WHO) recommends that TB drugs are administered by patients under supervision (DOT) (WHO, 2021). Although supervision by healthcare professionals is the best option, in some cases, such as in many developing countries, where resources are limited, this strategy is challenging to implement. In this case, the WHO advises that any person or video monitoring be used, as long as it is done in real time (WHO, 2020).

Studies have, however, identified the supervision by someone other than a healthcare professional (Woimo *et al.*, 2017, Fang *et al.*, 2019), including self-supervision (Zhou *et al.*, 2012) or a family member (Lei *et al.*, 2016, Lin *et al.*, 2017), as being a risk factor for LTFU. Conversely, one study, conducted in China, identified family supervision as a protective factor against treatment LTFU, owing to the general and psychological support offered by family members (Chen *et al.*, 2013).

### Treatment costs

Healthcare related costs, including direct treatment costs (Putera *et al.*, 2015, Lei *et al.*, 2016), and other indirect costs, such as lower income due to work disruption for treatment appointments (Chida *et al.*, 2015), have been identified as risk factors for TB treatment LTFU in Indonesia, China, and Pakistan, respectively. Although some countries provide free TB treatment as part of their national TB programs, these programs, such as the one in Ethiopia, was demonstrated to not necessarily include the medication required for the treatment of adverse drug events, such as liver damage, thereby adding to the probability of LTFU (Woimo *et al.*, 2017).

### Inadequate diagnostic regimes

Pre-treatment sputum smear results have been identified as a significant risk factor for TB treatment adherence. This relationship is, however, contradictory, with some studies linking a positive result to an increased chance of LTFU (Harling *et al.*, 2017, Afshari *et al.*, 2020), while others report that a negative result is more likely to result in LTFU (Flick *et al.*, 2016, Kigozi *et al.*, 2017, Muluye *et al.*, 2018), suggesting that the patients did perhaps not get adequate program attention, resulting in dissatisfaction with treatment services (Kigozi *et al.*, 2017).

TB re-treatment is another identified risk for treatment LTFU (Muture *et al.*, 2011, Culqui *et al.*, 2012, Finlay *et al.*, 2012, Garrido *et al.*, 2012, Marx *et al.*, 2012, Peltzer *et al.*, 2012, Chen *et al.*, 2013, Liddle *et al.*, 2013, Babiarz *et al.*, 2014, Cherkaoui *et al.*, 2014, Gafar *et al.*, 2014, Kigozi *et al.*, 2017, Wohlleben *et al.*, 2017, Mekonnen and Azagew, 2018, Muluye *et al.*, 2018, Mulongeni *et al.*, 2019, Ajema *et al.*, 2020, Ndambuki *et al.*, 2021), mainly because these patients are discouraged to go through a second round of treatment, which was unsuccessful the first time (Ajema *et al.*, 2020, Koo *et al.*, 2020). Before re-treatment commences, drug susceptibility testing is recommended to ensure the absence of any drug-resistant strains, which will require adapted treatment (Cadosch *et al.*, 2016). Bacteriological confirmation is necessary for drug susceptibility testing (WHO, 2021), but in 2020, only 59% of all global TB cases were bacteriologically confirmed, and merely 71% of these included rifampicin-resistance testing. By adding the lack of required infrastructure and questionable reliability of patient treatment history to the unconfirmed diagnosis, many re-treatment cases are being placed on the same treatment regime for a second time. This approach is against the WHO’s recommendation and is unlikely to result in a higher overall success rate, as shown in data recorded in West Ethiopia (Dedefo *et al.*, 2019).

### Treatment-related factors

Drug-susceptible, active TB is currently treated with a six-month DOT, short-course (DOTS) program (WHO, 2021). DOTS treatment consists of two phases, an initial, intensive two-month phase, which entails the co-administration of four first-line drugs, isoniazid, rifampicin, pyrazinamide, and ethambutol, followed by the second phase, including only rifampicin and isoniazid, for a further four months (WHO, 2021). It has been shown that the treatment burden (high pill load and long treatment period) as well as the occurrence of adverse drug events, could further contribute to LTFU.

### Treatment burden

The fact that these first-line drugs have to be administered daily, makes the pill burden of TB treatment too high for certain patients (minimum of 9 pills per day for a person weighing 50 kg (Blomberg *et al.*, 2001)), resulting in treatment non-adherence, as seen in populations from Ethiopia and Peru (Culqui *et al.*, 2012, Woimo *et al.*, 2017). In addition, treatment length is also recognized to be a barrier for treatment adherence, with some studies done in developing countries such as Ethiopia, Kiambu County, and India demonstrating that the majority of LTFU instances already occurred during the intensive phase of TB treatment (Mundra *et al.*, 2017, Woimo *et al.*, 2017, Kimani *et al.*, 2021). When systematically investigating the impact of different treatment lengths on treatment adherence and disease transmission in South Ethiopia, it was found that, while shorter treatment regimens may not necessarily reduce TB prevalence, the epidemiological advantages are closely correlated with improved treatment adherence (Pinho *et al.*, 2015).

### Adverse drug events

Many patients encounter adverse drug events during first-line TB treatment varying in degree of severity. A recent study from India found that 34% of all documented cases (n=545) under standardised DOTS treatment reported one or more adverse drug events (Imam *et al.*, 2020). Consequently, in 2015, another study conducted in the same country, found that a quarter of their study cohort reported LTFU due to adverse drug events, mostly within the first two months of treatment (Roy *et al.*, 2015). Substantiating this, is the fact several additional studies, done on cohorts from Ethiopia, India, Kenya, Morocco, Nigeria and Pakistan, also identified the experience of adverse drug events as a risk factor for treatment LTFU (Vijay *et al.*, 2010, Muture *et al.*, 2011, Culqui *et al.*, 2012, Finlay *et al.*, 2012, Sendagire *et al.*, 2012, Tachfouti *et al.*, 2012, Cherkaoui *et al.*, 2014, Chida *et al.*, 2015, Tesfahuneygn *et al.*, 2015, Wohlleben *et al.*, 2017, Woimo *et al.*, 2017, Gube *et al.*, 2018, Iweama *et al.*, 2021), which is in contrast to similar studies where no such significant association was found (Viegas *et al.*, 2017, Afshari *et al.*, 2020, Ajema *et al.*, 2020).

It is however important to note that other risk factors for treatment non-adherence, such as old age, anaemia, overweight, history of smoking, alcohol, and substance use, have all previously been associated with the increased occurrence of TB treatment adverse drug events (Chung-Delgado *et al.*, 2011). As a result, these factors might also be at play here, impacting the degree of adverse drug events experienced, and consequently also affecting TB treatment adherence.

### TB disease and other health-related factors

### TB disease severity

The severity of the TB disease and symptoms also has a probable influence on treatment adherence. Greater lung pathology (Jenkins *et al.*, 2013), severe cough (Nour El Din *et al.*, 2013), and TB-related morbidity (Theron *et al.*, 2015) at treatment commencement have all been associated with an increased likelihood of treatment LTFU. Furthermore, in Peru, low BMI (18.5kg/m**^2^**) at treatment enrolment was linked to TB treatment LTFU (Lackey *et al.*, 2015). This association, however, was shown to be insignificant in studies done in Rwanda (Kayigamba *et al.*, 2013) and Ethiopia (Muluye *et al.*, 2018).

### Comorbid health conditions

Numerous comorbidities with TB, including diabetes mellitus (Zhang *et al.*, 2014, Lackey *et al.*, 2015), HIV (Muture *et al.*, 2011, Garrido *et al.*, 2012, Jenkins *et al.*, 2013, Kayigamba *et al.*, 2013, Naidoo *et al.*, 2013, Lackey *et al.*, 2015, Nglazi *et al.*, 2015, Tesfahuneygn *et al.*, 2015, Harling *et al.*, 2017, Kigozi *et al.*, 2017, Silva *et al.*, 2017, Viegas *et al.*, 2017, Ambaw *et al.*, 2018, Mekonnen and Azagew, 2018, Muluye *et al.*, 2018, Mulongeni *et al.*, 2019, Iweama *et al.*, 2021, Workie *et al.*, 2021), cancer (Zhang *et al.*, 2014, Harling *et al.*, 2017) and depression (Peltzer *et al.*, 2012, Naidoo *et al.*, 2013, Theron *et al.*, 2015, Ambaw *et al.*, 2018), have been identified as risk factors for TB treatment LTFU in various developing countries.

The co-presentation of TB with HIV is, however, most commonly observed, and alarmingly, TB disease remains one of the leading causes of death among HIV-positive individuals (WHO, 2021). TB patients with an unknown or positive HIV status could be at risk for TB treatment LTFU if they are afraid of the HIV stigma or due to denial or unawareness regarding their HIV-status, as stated in a South African study (Kigozi *et al.*, 2017). On the contrary, an investigation in Ethiopia associated an HIV-negative status with a higher incidence of treatment LTFU, which was attributed to the enhanced counselling and support provided to HIV patients (Ambaw *et al.*, 2018). Similar findings were found in the low TB incidence, developed country, France, where the LTFU risk increased among non-HIV-infected TB patients (Tetart *et al.*, 2020).

In addition to the social factors, TB treatment, in combination with the existing treatment protocol of a comorbidity, is intolerable for some patients due to the extreme pill burden and adverse drug event occurrence (Kigozi *et al.*, 2017).

A high incidence of psychological distress (including symptoms of depression and anxiety) has been associated with TB patients in South Africa and these symptoms were identified as a contributing LTFU factor in this population (Theron *et al.*, 2015). Another study, conducted in Ethiopia (Ambaw *et al.*, 2018), found that 53.9% of their TB patient cohort had probable depression at treatment baseline. This resulted in a significantly higher LTFU rate at six months (3.9%), in contrast to those without baseline probable depression (0.8%). Ambaw *et al*. (2018) reasoned that depression may lead to poor self-care and substandard living conditions, which, in TB patients, can result in patients failing to take their prescribed medications and impede them to adhere to the treatment program *et al.*,.

### Practicality of interventions to improve TB treatment adherence in developing countries

Multiple intervention strategies have been proposed in an attempt to improve TB treatment adherence rates (WHO, 2003, National Department of Health, 2014). High rates of LTFU, nevertheless, persist, particularly in developing countries. In the following sections, we will provide an overview of more recently suggested intervention strategies, with particular application feasibility in developing countries.

### Socio-Economic support

As discussed earlier, socio-economic status has a significant impact on TB treatment adherence; hence, social protection is viewed as an essential component of TB control and eradication in the WHO’s End-TB Strategy (Baluku *et al.*, 2021, WHO, 2021). Previous studies conducted in developing countries have shown that social protection policies including cash or resource transfers improve TB treatment outcomes (Richterman *et al.*, 2018, Oliosi *et al.*, 2019).

One such initiative, launched in Uganda, was a one-dollar incentive during TB treatment (Baluku *et al.*, 2021). The aim of this initiative was to determine whether a small ‘affordable’ cash incentive (equivalent to one USD) would improve TB treatment outcomes in the most vulnerable populations. Baluku *et al*. (2021) reported that in rural Uganda, a clinic visit at a government institution for an individual with TB symptoms costs roughly one USD, which equates to about a quarter or more of these patients’ monthly household income. Accordingly, this study reported a 59% increase in the TB cure rate and a 56% reduction in treatment LTFU after providing the incentive (Baluku *et al.*, 2021).

Monetary incentives are further subdivided into conditional and unconditional. Conditional cash incentives require patients to meet particular behavioural, educational, or health requirements before getting the payment, whereas unconditional incentives do not (Boccia *et al*. 2016). Richterman *et al*. (2018) compared the benefits of conditional and unconditional cash incentives in health systems and discovered that conditional transfers have positive effects on general health behaviour and outcomes, whereas the effects of unconditional cash transfers were still uncertain.

Additionally, since TB treatment requires a lengthy treatment period, conditional cash transfers were suggested to be more appropriate as a TB treatment adherence intervention (Richterman *et al.*, 2018). This is substantiated by the Ugandan one-dollar incentive, which was solely conditional on patients turning up for their clinic appointments, which as previously stated, resulted in improved treatment outcomes (Baluku *et al.*, 2021). Although past studies have also demonstrated that incentives increase TB successful treatment outcomes, these incentives were often too high to be viable standardised practice in resource-limited, developing countries (Ukwaja *et al.*, 2017, Wingfield *et al.*, 2017, Richterman *et al.*, 2018). Another reported risk of this intervention is that, if the monetary incentive is too high, it may encourage patients who are desperately in need of money to remain ill. As a result, the one-dollar incentive proposed in Uganda (Baluku *et al.*, 2021), in which the money reward principally covers the expenditures incurred during clinic visits, is suggested to be more feasible.

### Healthcare and patient-related interventions

Since treatment illiteracy or insufficient pre-treatment counselling are determined here as LTFU contributing factors, the enhancement of such practices is one logical intervention option. Interestingly, however, Cherkaoui *et al*. (2014) highlighted that previous adherence interventions focusing on single-factorial patient-related or healthcare system-related determinants, such as general TB education incentives, have shown minimal to marginal benefits in developing countries in the past, with more success reported when combining these strategies.*et al.*,

Accordingly, it was suggested that treatment adherence interventions could benefit more by addressing gaps within both patient-related and healthcare system-related determinants of LTFU (Cherkaoui *et al.*, 2014). A few of these previously recommended strategies are reiterated here. Not only, is it imperative for DOT providers to establish and maintain a good patient-provider relationship, but attention should also be given to appropriate patient follow-up, the treatment or prevention of adverse drug events, and to increase the number of decentralised healthcare facilities (especially in rural areas) (Finlay *et al.*, 2012, Cherkaoui *et al.*, 2014, Roy *et al.*, 2015). The rationale behind the lengthy treatment and the necessity of treatment completion despite TB symptoms improvement or the occurrence of adverse drug events, should be discussed (Roy *et al.*, 2015, Ahmed and Mohan, 2021). Additionally, any doubts regarding the disease and treatment should be explained before treatment onset and continuously emphasised during treatment (Roy *et al.*, 2015, Ahmed and Mohan, 2021). Furthermore, the necessary information regarding the options available to obtain medication during travelling should be provided (Muture *et al.*, 2011).

To further assist good provider-patient relations, it was suggested that enhanced communication and counselling training for healthcare workers along with the use of appropriate patient education materials, be implemented (Finlay *et al.*, 2012). Further program supervision and management improvement were suggested (Finlay *et al.*, 2012) and with frequent monitoring of minor adverse drug events, patients could be treated, reassured, or advised on the correct drug administration procedures (Roy *et al.*, 2015), which in turn could prevent them from treatment LTFU.

Combined psychological counselling and adherence education throughout treatment have been shown to reduce treatment non-adherence in Ethiopia (Tola *et al.*, 2016). Furthermore, in India, four individual counselling sessions done by highly skilled interventionists at months 0, 2, 4, and 6 during TB treatment, were determined to be a viable treatment adherence intervention in TB patients with alcohol dependence (Thomas *et al.*, 2017). The patients undergoing counselling sessions showed significantly higher treatment adherence than those who did not, and the intervention group also had significantly lower Alcohol Use Disorder Identification Test (AUDIT)-scores after treatment completion, relative to that measured before therapy commenced (Thomas *et al.*, 2017).

One intervention strategy that has shown some promise in improving treatment adherence in both developed and developing countries, is the use of mobile health technologies (such as text messaging, phone calls and video observed treatment) to remind and monitor drug administration (Fang *et al.*, 2017, Alipanah *et al.*, 2018, Garfein and Doshi, 2019, Kruse *et al.*, 2019). Cross *et al*. (2019) reported one such example of an alternative low-cost adherence tracking approach, incorporated in India’s standard TB treatment program for the past few years, 99DOTS. Here, the dispensing of pills can be tracked through the packaging, which contains a unique code for each drug dose. Once opened, this code must be sent by the patient to the healthcare worker via a telephone call (Cross *et al.*, 2019). 99DOTS further provides healthcare workers with real-time information, allowing them to monitor patients’ adherence to treatment and notify them of missed doses (Cross *et al.*, 2019, Thakkar *et al.*, 2019). A reported drawback of this method, however, is that the patient should have regular access to a telephone, which might be more difficult in other resource-constrained developing countries (Cross *et al.*, 2019). Other researchers in India, further suggested that improved implementation strategies are required, as some patients currently make the calls without ingesting the drugs, consequently affecting the accuracy of adherence reporting (Thomas *et al.*, 2020).

As mentioned, another key risk factor for TB treatment LTFU is the challenge of physically getting to TB healthcare facilities. A few strategies have been evaluated over the years to address this issue. For example, in 2019, a study conducted in Saudi Arabia, which is classified as a developed country, compared the LTFU rate among TB patients who were treated using a facility-based DOTS venue versus those treated with a newly proposed Community Mobile Outreach Approach (AlSahafi *et al.*, 2019). The use of the mobile centre resulted in a much lower LTFU rate (3 % vs. 22 % for the facility-based treatment) for the duration of this study, and the mobile outreach also resulted in patients having a better understanding of TB and its treatment (AlSahafi *et al.*, 2019). AlSahafi *et al*. (2019) further stated that this technique has, similarly, been efficiently implemented in some developing countries, including Myanmar and Pakistan, and its success can be attributed in part to patient satisfaction. This approach, however, is stated to be more difficult to implement in countries where TB stigmatisation is prevalent, because TB drugs are delivered directly to a patient’s doorstep (AlSahafi *et al.*, 2019). In this case greater emphasis should be put on improving TB knowledge and eliminating the stigma associated with this infectious disease.

## Conclusion

TB treatment efficiency is dependent on the timely initiation and continuation of treatment. This could prevent patient prolonged illness and the development of clinical complications, drug resistance, or premature death (Kigozi *et al.*, 2017). Furthermore, treatment adherence also hinders TB transmission, since patients should become less infectious within two weeks of adequate treatment (Kigozi *et al.*, 2017).

TB treatment LTFU is a major obstacle in the global goal of ending the TB epidemic by 2030 (WHO, 2021). Through this overview, a summary of factors contributing to treatment LTFU was provided in order to promote awareness of this ongoing challenge in TB treatment, specifically in developing countries. It is clear that the LTFU risk factors identified previously (WHO, 2003, National Department of Health, 2014) still persist in developing countries in 2022, and that newer interventions or better adherence and monitoring to interventions, need to be implemented urgently.

It is worth noting, however, that the factors discussed above could only be recognized as possible risks for TB treatment LTFU if it has’ been investigated and included as a possible variable in the specific study. Hence, the absence of some factors in certain investigations isn’t always attributable to a lack of significance, but rather to the study experimental design.

Several adherence strategies were also discussed. Patient-provider interactions and communication should be prioritised, since a thorough understanding of what TB is and how the treatment works can assist in overall treatment adherence rates. Alternative strategies, such as the above-mentioned mobile outreach teams or monetary incentives, may be worth exploring in developing countries.

### Statement on Conflict of Interest

The authors declare that there are no competing interests associated with this study.

**List of Abbreviations:**
AIDS: Acquired immune deficiency syndrome;
AUDIT: Alcohol Use Disorder Identification Test;
DOT: Directly observed treatment;
DOTS: Directly observed treatment, short-course;
HIV: Human immunodeficiency virus;
LTFU: Lost to follow-up;
*M. tuberculosis:*
*Mycobacterium tuberculosis*;
TB: Tuberculosis
WHO: World Health Organisation;
